# The Perils of Complementary Alternative Medicine

**DOI:** 10.5041/RMMJ.10153

**Published:** 2014-07-25

**Authors:** Michael J. Bayme, Alex Geftler, Uri Netz, Boris Kirshtein, Yair Glazer, Shahar Atias, Zvi Perry

**Affiliations:** 1 Surgery Ward A, Soroka University Medical Center, Beer Sheva, Israel;; 2 Orthopedic Ward, Soroka University Medical Center, Beer Sheva, Israel and; 3 Center for Medical Education, Ben-Gurion University, Beer Sheva, Israel

**Keywords:** Complementary alternative medicine, formal legislation, group B streptococcus, spontaneous spinous epidural hematoma

## Abstract

More than 11,000 articles lauding alternative medicine appear in the PubMed database, but there are only a few articles describing the complications of such care. Two patients suffering from complications of alternative medicine were treated in our hospital: one patient developed necrotizing fasciitis after acupuncture, and the second developed an epidural hematoma after chiropractic manipulation. These complications serve as a clarion call to the Israeli Health Ministry, as well as to health ministries around the world, to include complementary medicine under its inspection and legislative authority.

## INTRODUCTION

There is no better way to exemplify the spread of alternative medicine than to perform a search of the shrine of formal medicine—PubMed. Typing the words “complementary alternative medicine,” or the popular acronym CAM, brings up more than 11,000 articles. However, searching for “complementary alternative medicine (CAM) safety” yields only 800 articles, and hits related to complications result in only 20 articles! Is this because we do not take CAM seriously enough and therefore do not consider its dangers, thereby exposing patients to unlicensed and/or partially trained practitioners?

Using two cases from our hospital, this paper illustrates and discusses the risks involved with complementary medicine—risks that mandate health authorities such as the Israeli Ministry of Health to include complementary medicine under its inspection and legislative authority. By doing so, we believe that complementary medicine will be safer for patients—the goal of all traditional or alternative health care providers.

## CASE OVERVIEWS

Two patients suffering from complications of alternative medicine were treated in our hospital: one patient developed necrotizing fasciitis after acupuncture, and the second developed an epidural hematoma after chiropractic manipulation.

### Case I

The first case is that of a 59-year-old male, who underwent a course of acupuncture for chronic low back pain, by a young female acupuncturist. During the therapy the patient noted swelling at the point of puncture, but his therapist dismissed the claim. The region continued to swell, and three days later the patient presented to his family doctor, who diagnosed cellulitis and prescribed oral amoxicillin with clavulanic acid (Augmentin; GlaxoSmithKline plc, Brentford, UK). The following day the patient’s condition worsened—he started to suffer from chills and more intense pain, so he went to the emergency room.

**Figure 1.**
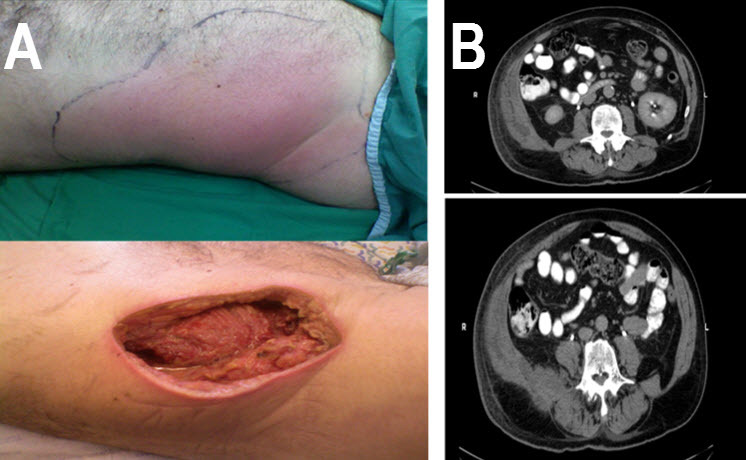
**Photo and CT Scan of the Patient’s Right Flank.** A: Right flank before and after surgery; B: CT scan of the afflicted area.

Upon examination, the patient had a fever of 37.9°C, a pulse of 119, and a blood pressure of 199/87. Edema was noted over the patient’s entire right flank (Figure 1A). Laboratory results were notable for a level of glucose of 298 mg/dL, sodium of 128 mmol/L, and white blood count (WBC) of 26,500 cells/μL with left shift. An emergency CT revealed an abscess of the abdominal wall involving the muscles, but no intra-abdominal pathology (Figure 1B).

The patient received broad-spectrum antibiotics and was taken to the operating room for debridement. Upon incision there was subcutaneous edema with no puss, gangrene of the entire external oblique muscle, and an abscess between the external and internal oblique muscles. The muscles were debrided back to healthy, bleeding tissue and the wound copiously irrigated with saline. The wound was left open, with gauze and iodine as a cover. Gram stains and cultures returned group B streptococcus (GBS) sensitive to penicillin, and antibiotic coverage was adjusted accordingly. The patient returned to the operating room for serial debridement until the wound developed healthy granulation tissue, as is well advocated in the medical literature.^1^–^6^ The patient received four units of blood and required 13 days of hospitalization. To date, he suffers from a disfiguring wound of his abdominal wall.

Considering the fact that group B streptococci live primarily in the female vagina, and that the acupuncturist was a young female, it is possible to assume that the cause for this grave illness was due to improper hygiene while treating our patient with acupuncture. Although rare, this tragic consequence of acupuncture has been seen previously by other researchers.^7^ Research has also shown that GBS has been transmitted via person-to-person direct contact; thus, an association between GBS colonization and hand-washing in the general population seems likely. The crude association of tampon use with GBS (OR, 5.7) was statistically significant, which also strengthens our suspicion of transmission from the female acupuncturist.^8^

### Case 2

**Figure 2.**
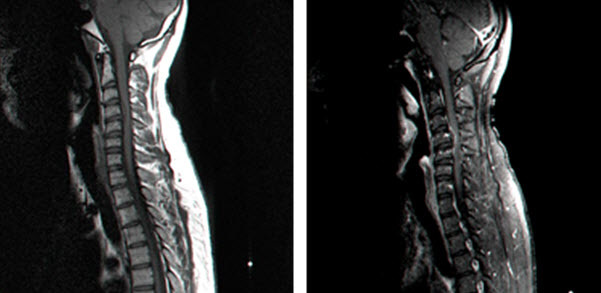
**MRI Imaging after Surgery.** Left: T1-sagittal epidural hematoma at C3-4 level; Right: T1 STIF sagittal epidural hematoma at C3-4 level.

The second case involved a 27-year-old male with chronic cervical pain, without any previous medical treatment or imaging, who was referred to our tertiary medical facility. The patient suffered from back pain. To manage his pain, the patient used the services of a chiropractor who used cervical manipulation. Immediately after such a manipulation, the patient felt a severe cervical pain; 30 minutes after manipulation the patient started feeling paresthesia in his hands and legs. The patient was admitted to our emergency room with symptoms of progressive weakness in all four extremities and weakness that was measured as 3/5. No additional symptoms were seen. Immediate MRI demonstrated an epidural hematoma at the C3-4 level (Figure 2).

The patient underwent immediate surgery to evacuate the hematoma via an anterior approach and C3-4 cage placement. The day after surgery the patient showed a remission of symptoms. At 6 months follow-up his remission was complete.

The incidence of spontaneous spinous epidural hematoma (SSPE) is considered to be very low. Kuker et al. reported that SSPEs most frequently occur at the thoracic or lumbar spine, with incidences in the cervical region being quite rare—only 1 out of 7 reported cases.^9^ The hematoma can be epidural or subdural. The presenting symptoms of SSPE may be shoulder pain,^10^ interscapular pain,^11^ associated with radicular radiation into the upper extremities, neck pain,^12^ hemiparesis like in cases of Brown–Sequard syndrome,^10^,^11^,^13^,^14^ progressive weakness,^12^ or even spinal shock.^15^ Symptoms of neck and upper extremity pain with bilateral signs of myelopathy with a sensory level should lead to the suspicion of acute cervical cord compression.^16^ A few case reports have shown that SSPE can follow minor traumatic injury as in a motor vehicle accident.^17^ To our knowledge, the literature includes only three reports of SSPE immediately following a chiropractic manipulation that was considered the cause of this event.^17^–^19^ We conclude from the current case that chiropractic procedures can be dangerous when performed by practitioners who might be only partially trained, who might tend to perform an insufficient patient examination before the procedure, and thus endanger their patients. In the current case, an acute epidural hematoma diagnosed by immediate MRI followed by emergency decompression within six hours of presentation resulted in complete recovery. We believe that the standard treatment in these cases is a prompt surgical evacuation of the hematoma, after MRI as the imaging modality, as had been done in the current case.

## DISCUSSION

The above two cases have not been shared in order to torment or scold those who work as CAM providers or who use CAM services. Both cases resulted in grave consequences, which fortunately did not end in death or paraplegia.

One can certainly find such problematic consequences in traditional medicine, but with a significant difference—*all* traditional medical providers are taught in, and practice under, severe surveillance in an environment conducive to damage control. This means that physicians are monitored, taught, and seek for ways to prevent adverse effects to their patients. We are sure that CAM providers have the same good intentions, but they lack any formal monitoring—neither in their training, nor in their daily activity. If one looks at the web site of the Israeli Ministry of Health one can find thorough details regarding the number of physicians, dieticians, physiotherapists, etc. However, there are no data whatsoever (not even an estimate) for CAM providers. In the same web site one can learn that the Ministry of Health has proposed a law regarding the professional status of chiropractors in 2010, but nothing has been implemented since then. This means that the young lady who caused the severe necrotizing fasciitis due to poor hand hygiene has no idea that she caused such a grave mishap; neither can she be reprimanded since there is no formal legislation or monitoring of her activity. The same applies for the chiropractor who caused his patient to suffer from a SSPE. He was notified of the problem, but since his profession has no real legal standing in Israel, notification of the incident to the Ministry of Health would have been ineffective as there was no Israeli-issued license that could be revoked. One must remember that acupuncture and chiropractice are the most studied CAM therapies with many control studies that have also reported side effects. Even so, when searching PubMed, one can find more than 1,800 articles about chiropractic manipulation, but only three discuss the dangers in these manipulations.^20^–^22^ Some leading Israeli hospitals include CAM in their services, mainly in pain clinics. The health insurance programs also include such services under their umbrella. Some Israeli schools for CAM therapies offer four years of training. However, no real state monitoring, surveillance, or mentoring programs have been established for those who work in the field.

Considering all of the above, one cannot overlook the issue of CAM and its perils, even if legislation means a considerable amount of money. Both traditional and CAM health care providers must insist on formal legislation—we cannot afford to sit back and hope for the best. Even the British Medical Journal has recognized this in an editorial, *Acupuncture is safe in the hands of competent practitioners*.^23^ The title alone implies that there are those who are not competent acupuncturists. However, who has the responsibility of determining who is a competent acupuncturist? Nobody knows, and nobody in Israel seems to have a proper answer for this question.

These issues are not minor Israeli-based problems, but they engulf medical professionals all over the world. Cohen et al. have shown that these problems are unmet in the field of psychiatry in the USA,^24^ just as George and Iyer^25^ have pondered these issues for rural India. Wang et al.^26^ have tried to understand why acupuncture is flourishing in the USA as it is in China, and they also state that legislation in this area is lacking in the USA. Senzon^27^ embraces this call to legislation and believes that only a major reorganization of the American health care system and its attitude towards CAM can make the system an efficient and effective one. These cases call for a fundamental change in our attitude, as well as in the demands we should place on our fellow CAM providers. Complementary alternative medicine should no longer be a semi-legal profession open for exploitation. The Ministry of Health should endorse a formal curriculum, as is done with other medical professions, which is strictly enforced. People who train in CAM should be formally evaluated by the Ministry of Health, and there should be legal and financial sanctions against those who will not obey these rules—all in the same manner as for any other formal health care provider.

On the other hand, those who obey these rules should be able to be a part of the formal health care system, or at least of the complimentary health insurance, just as they are today.

We all know mistakes happen to every physician in the hospital. This fact alone should cause CAM therapists themselves to call for a change. Without a legal standing, professional indemnity insurance may be limited or unavailable, making the practitioner totally liable in the event of an unfortunate mistake.

Health ministry officials need to include in such a curriculum hygiene and safety issues, in order to improve the CAM providers’ patient treatments. A formal professional surveillance system needs to be implemented, similar to that of traditional health care providers. Taking action with regard to all these measures will help us all provide for a safer, healthier, and more effective health care system—whether it utilizes traditional or alternative medicine.

## Abbreviations:

CAM: complementary alternative medicine
GBS: group B streptococcus
SSPE: spontaneous spinous epidural hematoma
WBC: white blood count
